# Stem cells redirect the neuroinflammatory trajectory of primed microglia in an ovine chorioamnionitis model

**DOI:** 10.1016/j.bbih.2026.101359

**Published:** 2026-09-18

**Authors:** Valéry L.E. van Bruggen, Sanne J.C.M. Claassen, Luise Klein, Cindy Bokobza, Jennifer Hua, Tiphaine Marvillet, Michelle M.E. Zandbergen, Charlotte van Gorp, Florian Caimant, Renate G. van der Molen, Pierre Gressens, Daan R.M.G. Ophelders, Tim G.A.M. Wolfs

**Affiliations:** a Department of Pediatrics, Maastricht University Medical Center+, MosaKids Children's Hospital, Maastricht, AZ, 6202, the Netherlands; b GROW–Research Institute for Oncology and Reproduction, Maastricht University Medical Center, Maastricht, ER, 6229, the Netherlands; c Université Paris Cité, Inserm, NeuroDiderot, Paris, F-75019, France; d GenomiqueENS, Institut de Biologie de l’ENS (IBENS), Départment de biologie, École normale supérieure, CNRS, INSERM, Université PSL, Paris, 75005, France; e Erasmus MC, University Medical Center Rotterdam, Department of Public Health, Rotterdam, 3015 GD, the Netherlands; f Department of Toxicogenomics, Maastricht University, Maastricht, 6229 ER, the Netherlands; g Department of Laboratory Medicine, Laboratory of Medical Immunology, Radboud University Medical Center Nijmegen, the Netherlands

**Keywords:** Ovine model, Chorioamnionitis, Neuroinflammation, Preterm brain injury, Microglia, Stem cell therapy

## Abstract

Antenatal infection (e.g. chorioamnionitis), an important risk factor for preterm birth, can induce a fetal systemic inflammatory response (FIRS), initiating neuroinflammation. Microglia, as principal resident immune cells of the brain, are key targets for therapeutic interventions such as Multipotent adult progenitor cells (MAPC). We analyzed the effects of intravenous MAPC therapy on microglia and peripheral immune cells at the blood-brain barrier (BBB) in an ovine chorioamnionitis model.

Ovine fetuses were intra-amniotic (i.a.) exposed to lipopolysaccharide (LPS) to induce FIRS, or saline (SAL) at 125 days gestational age (dGA), followed by intravenous MAPC therapy or SAL at 127 dGA. After sacrifice (132 dGA), isolated microglia were analyzed by qRT-PCR and RNA sequencing. Microglial function was assessed through efferocytosis of Jurkat cells. Additionally, immunohistochemistry of immune cells (CD45, IBA-1, MPO) and their ANXA1 co-expression were quantified in brain parenchyma and BBB.

Antenatal inflammation resulted in an increased efferocytotic capacity following *in vitro* LPS re-exposure and gene ontology annotations revealed significant changes in immunomodulatory genes, indicating microglial priming. This was further confirmed through selective upregulation of IL-10 and ANXA1, accompanied by increased COX-2 in MAPC-treated animals following i.a. LPS exposure. This was paralleled by increased numbers of ANXA1+ immune cells at the BBB and brain parenchyma. Microglial RNA transcriptomic analysis underlined the immunomodulatory and postulated neuroprotective effects of MAPC therapy.

We conclude that antenatal inflammation primes microglia which can be targeted by MAPC therapy. Early redirection of neuroinflammation may preserve neural integrity and prevent subsequent neurocognitive impairments in later life.

## Highlights

• Antenatal LPS exposure primes microglial function and transcriptome.
• MAPC therapy targets peripheral immune cells in the course of antenatal inflammation.
• Early MAPC therapy attenuates pro-inflammatory microglial activation and redirects microglia toward a more immunomodulatory phenotype

## Graphical abstract

Intravenous stem cell administration after intra-amniotic LPS exposure of fetal sheep leads to an accumulation of immune cells expressing ANXA1 at the blood-brain barriers and in brain parenchyma. Immunomodulatory genes are upregulated in microglia upon stem cell treatment, increasing cytokine expression while maintaining efferocytotic capacity. Abbreviations: ANXA1; annexin A1, IL-10; interleukin 10, COX-2; cyclo-oxygenase 2, IL-8; interleukin 8, LPS; lipopolysaccharide. Figure created with BioRender.com.

**Figure:**
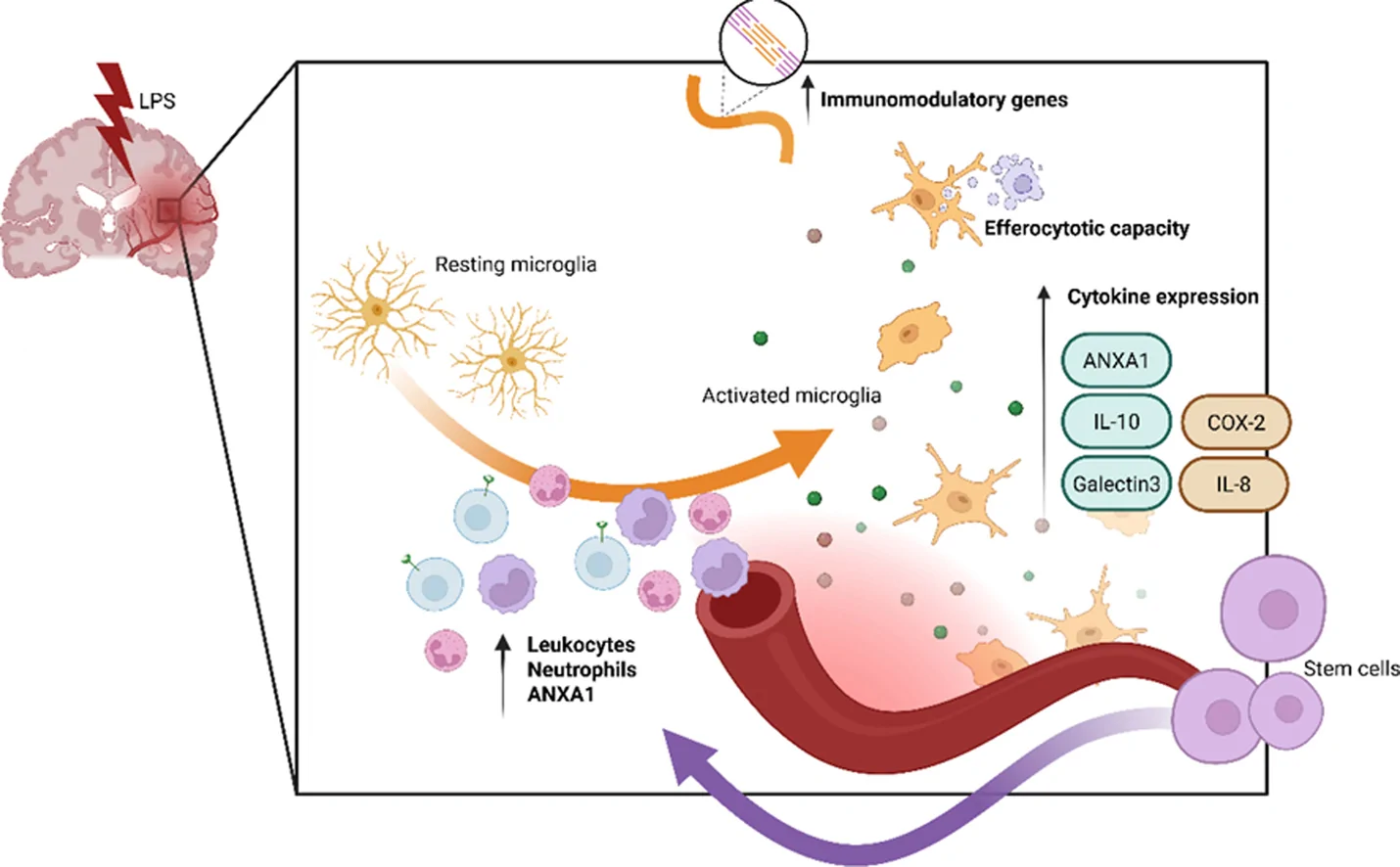

## 1 Introduction

Risk factors for preterm birth and subsequent neonatal mortality and morbidity, include intrauterine infections (e.g. chorioamnionitis) and concomitant systemic inflammation (Gussenhoven et al., 2018; Gotsch et al., 2007; Paton et al., 2017a). Systemic inflammation in the fetus affects the brain via the blood-brain barrier (BBB), by promoting persistent neuroinflammation leading to poor neurodevelopmental outcomes in former preterm born infants (Paton et al., 2017a; Kallapur et al., 2014; Lu et al., 2016).

The BBB is a complex barrier included in the neurovascular unit (NVU), consisting of brain endothelial cells, basal lamina and glial cells including microglia. Through the BBB, circulating peripheral immune cells influence cerebral immune homeostasis by producing immune mediators (Iadecola, 2017). Simultaneously, peripheral immune cells such as macrophages migrate into the brain parenchyma through the BBB, which serves as a regulatory gateway for these cells (Shechter et al., 2013). This migration may occur through tight junctions of the BBB, which can become permeable due to neuroinflammation (Yan et al., 2004; Stolp et al., 2005).

Microglia, the resident innate immune cells in the central nervous system (CNS), are essential in transducing systemic inflammation into neuroinflammation (Fleiss et al., 2021). Microglia respond to microenvironmental cues through their large repertoire of pattern recognition receptors that orchestrate processes to restore brain homeostasis after perturbation. Phagocytosis, vasculogenesis and neurogenesis play vital roles in brain development (Mallard et al., 2019; McNamara and Miron, 2020). These processes are essential for brain development and can be dysregulated during neuroinflammation, mediating brain injury and could ultimately lead towards long-term brain damage. Systemic inflammation caused by perinatal stressors such as infection or hypoxic-ischemia, induces a microglia phenotype characterized by the release of reactive oxygen species and pro-inflammatory cytokines (Fleiss et al., 2021). The acute pro-inflammatory response is ideally followed by an anti-inflammatory, reparative phase to ensure normal brain development (Fleiss et al., 2021). If the balance between pro- and anti-inflammatory microglia are not appropriately regulated, unresolved inflammation perpetuates into chronic neuroinflammation, which can persist for weeks and even years, causing more damage (Fleiss et al., 2021). Microglia are highly dynamic and phenotypic changes depend, among others, on the timing, duration and location of injurious events (van der Poel et al., 2019; Tan et al., 2020). Characterizing the phenotype of microglia in a disease model helps to understand the etiology of inflammation-induced brain injury and supports screening the efficacy of therapeutic interventions. Microglia are essential for tissue repair in neonatal brain injury, and their complete suppression or ablation has been shown to deteriorate brain outcome on the long-term (Tsuji et al., 2020; Chhor et al., 2017). Thus, modulation of their inflammatory response rather than suppression seems to be a promising approach. Simultaneously, the contribution of peripheral monocytes and neutrophils to microglial function in neuroinflammation and its resolution is underestimated (Chen et al., 2020; Liu et al., 2018; Richter et al., 2025).

Peripheral immune cells contribute to the resolution of inflammation by transferring proresolving mediators into tissues such as Annexin A1 (ANXA1). ANXA1 is recognized as down-stream effector of glucocorticoids and mediator of resolving inflammation and is highly expressed in neutrophils and monocytes/macrophages (Perretti and D'Acquisto, 2009). Furthermore, ANXA1 has been identified as a downstream effector of stem cell-based therapies and is essential for mediating their therapeutic efficacy (Gussenhoven et al., 2019; Rackham et al., 2016; Zagoura et al., 2019). Among others, this constitutes a rationale for stem cells as a promising therapy for systemic inflammation-induced brain injury. Intravenous administration of stem cells or stem cell-based therapies have been shown to be neuroprotective in the context of fetal hypoxic ischemic brain injury and improved functional and structural brain outcomes (Jellema et al., 2013, 2015; Ophelders et al., 2016; Malhotra et al., 2023; Kaminski et al., 2020). Specifically, multipotent adult progenitor cells (MAPCs) have potential due to their immunomodulatory and regenerative capacities (Passera et al., 2021; Yates et al., 2021; Le Blanc and Mougiakakos, 2012). There is accumulating evidence that MAPC effects, amongst others, are mediated by modulation of the peripheral immune response and microglia activation potentially involving cross-talk between stem cells and macrophages which might be paracrine, endocrine or via direct signaling (Jellema et al., 2013, 2015; Ravanidis et al., 2017; Walker et al., 2012). The ovine model was selected because it is a well-established and clinically relevant large-animal model that closely resembles human fetal development, particularly with respect to brain maturation and white matter development, while its frequent singleton pregnancies, suitability for chronic fetal instrumentation, and comparable size to human infants enable longitudinal physiological monitoring and evaluation of clinically relevant therapeutic interventions.

This study explored the neuroimmune response in a preclinical ovine model for chorioamnionitis through comprehensive analyses of peripheral immune cells at the blood-brain barrier and microglial function and transcriptome. Within this model, the effects of MAPCs therapy on the neuroimmune response were evaluated.

## 2 Methods

### 2.1 Study approval

Experimental procedures and the study design were approved by the Central Authority for Scientific Procedures on Animals (AVD107002016784) and in line with institutional guidelines for animal experiments of the Animal Welfare Body of Maastricht University, The Netherlands. This study was conducted following the ARRIVE guidelines for reporting animal research (Percie du Sert et al., 2020).

### 2.2 Experimental design and animals

27 Texel ewes were randomized into four groups (n = 5 to 6/group (Gussenhoven et al., 2018): intra-amniotic (i.a.) saline and intravenous (i.v.) saline treatment (SAL-SAL) (Gotsch et al., 2007), i.a. 5 mg lipopolysaccharide (LPS, O55:B5; L2880, Sigma-Aldrich, Zwijndrecht, NL; 5 mg) and i.v. saline treatment (LPS-SAL) (Paton et al., 2017a), i.a. saline and i.v. MAPC therapy (SAL-MAPC), and (Kallapur et al., 2014) LPS and MAPC therapy (LPS-MAPC) (Fig. 1). On gestational age (GA) 131 and GA 132, a total of four ewes spontaneously delivered premature (SAL-MAPC n = 2, LPS-SAL n = 2), which was an exclusion criterion.

**Fig. 1:**
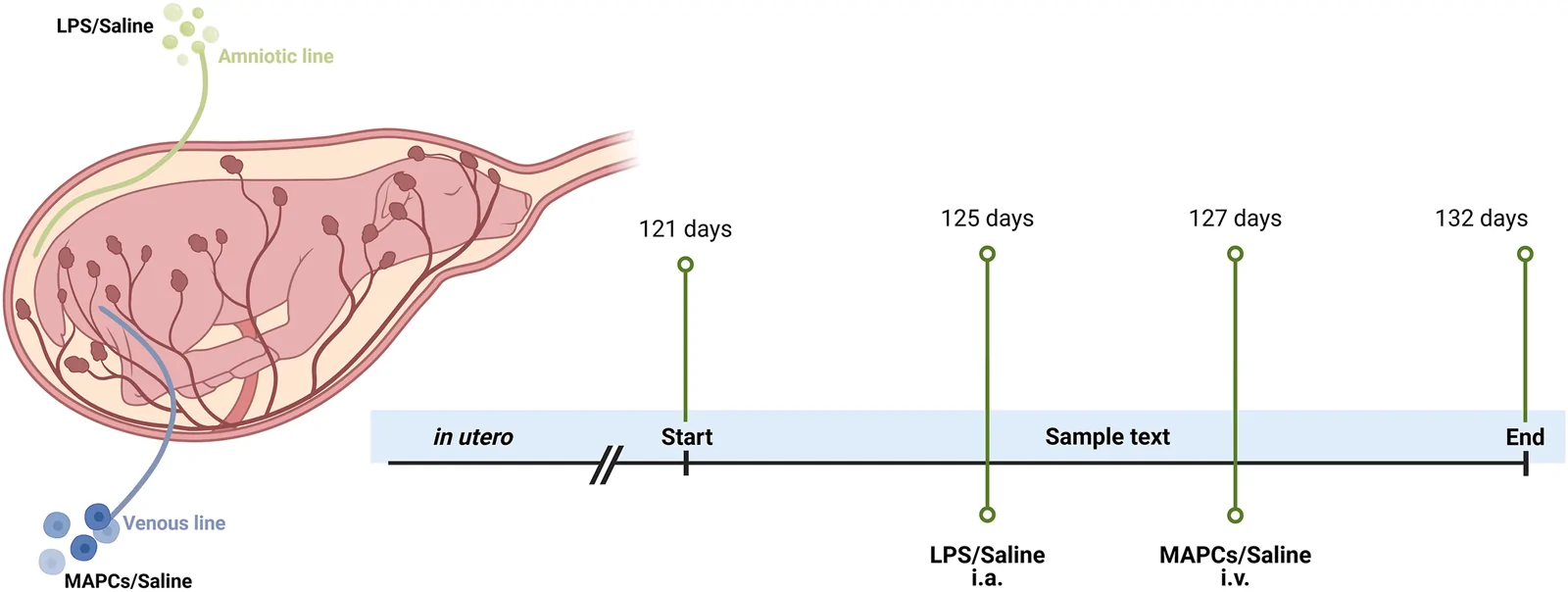
**Experimental design.** Pregnant ewes were instrumented on day 121 of gestation to receive an intra-amniotic injection of LPS/SAL at day 125 of GA and an intravenous injection of MAPC therapy/SAL at 127 of dGA. Fetuses were delivered at 132 dGA. Abbreviations: GA; gestational age, i.a.; intra-amniotic, i.v.; intravenous, LPS; lipopolysaccharide, MAPC; multipotent adult progenitor cells, SAL; saline. Figure created with BioRender.com.

### 2.3 Surgery and monitoring

Fetuses of time-mated Texel ewes were instrumented at 121 days of GA as previously described with minor adjustments (Jellema et al., 2013; Nikiforou et al., 2015). 121 GA in sheep (full term is approximately 147 days) is comparable to moderate/late preterm neonates, with regard to brain development (Back et al., 2012). Ewes were anesthetized by intravenous induction of thiopental (15 mg/kg). During surgery, general anesthesia was maintained by 1% to 2% isoflurane and followed by remifentanil (0.75 μg kg^−1^ min^−1^) i.v. for analgesia and local anesthesia at the wound site. A catheter was introduced to the maternal saphenous vein to obtain blood samples and administer prophylactic antibiotics (amoxicillin clavulanic acid) during 4 days of recovery after surgery. Fetuses were exposed by median laparotomy, and two 5 French umbilical vessel catheters (Covidien, Mansfield, MA) were placed in the femoral artery and vein for blood pressure recordings, blood sampling, and administration of stem cells. An additional catheter was placed in the amniotic sac for recordings of amniotic pressure and administration of LPS. All leads were exteriorized through a trocar hole in the maternal flank of the ewe and after closing the laparotomy, lines were continuously flushed (except from the amniotic catheter) with heparinized saline (25 IU/mL, 0.2 mL/h). Postoperative analgesia of the ewes consisted of flunixin meglumine administered for at least 2 days, after which buprenorphine was used if continued analgesic treatment as necessary.

while being housed in confined space with access to food and water ad libitum. Fetal blood was taken every morning to monitor the fetal systemic immune response after i.a. LPS administration at 125 days of gestation and i.v. administration of 10*10^6^ bone marrow derived MAPCs (Athersys Inc. (Cleveland, Ohio, USA)/Healios K.K. (Tokyo, Japan)) at day 127 GA. Plasma was isolated from fetal blood and stores and −80°C until further analysis. Systemic immune response was evaluated in previous studies by measuring circulating fetal IL-6 levels using ELISA (Klein et al., 2022).

### 2.4 Multipotent adult progenitor cells

Bone marrow-derived cells were isolated and expanded using the Quantum Cell Expansion System (Terumo BCT, Lakewood, CO) as described (Cunha et al., 2017) in off-the-shelf xenobiotic free media supplemented with additional MAPC growth factors, cultured under low oxygen conditions, and cryopreserved in liquid nitrogen gas phase until use. The cell product was manufactured as an off-the-shelf, readily available therapy. Cells were phenotyped in terms of marker expression and differentiation potential, as described before (Crabbé et al., 2016). MAPCs (10 × 10^6^ cells/mL) were thawed at room temperature for maximally 1 h and sterile administered in a single 1 mL bolus via the femoral vein to the fetuses (127 gestational age) randomized to MAPC therapy. Animals randomized to saline groups received equal amount of 0.9% saline injection at the same time point. Steroids (Celestone, Schering Plough Labo NV, Heist-op-den-Berg, Belgium) were intramuscular administered to the ewes 24 h before birth. On day 132 of gestation, all fetuses were surgically delivered by C-section and immediately euthanized with intravenous injected pentobarbitone (100 mg/kg).

### 2.5 End of experiment and brain processing

Fetal brains were harvested and halved along the midline and each hemisphere was further dissected coronally into 5 mm-thick blocks. Blocks from the left cerebral hemisphere were used for microglia isolation. Blocks from the right cerebral hemisphere was immersion-fixed in 4% paraformaldehyde in 0.1 M phosphate buffer (pH 7.4), processed through alcohol and xylene washes and embedded in paraffin wax. Equivalent blocks, at three different levels (frontal cortex, striatum, and mid-thalamus) were chosen from the right hemisphere of each animal for subsequent molecular and immunohistochemical analysis (Fig. 2). Immune cells in the brain parenchyma were counted in predefined regions (frontal cortex, striatum, and mid-thalamic region including lateral ventricles). To assess immune cells at the BBB in all three regions, cerebral blood vessels were selected as described before (Park et al., 2023).

**Fig. 2:**
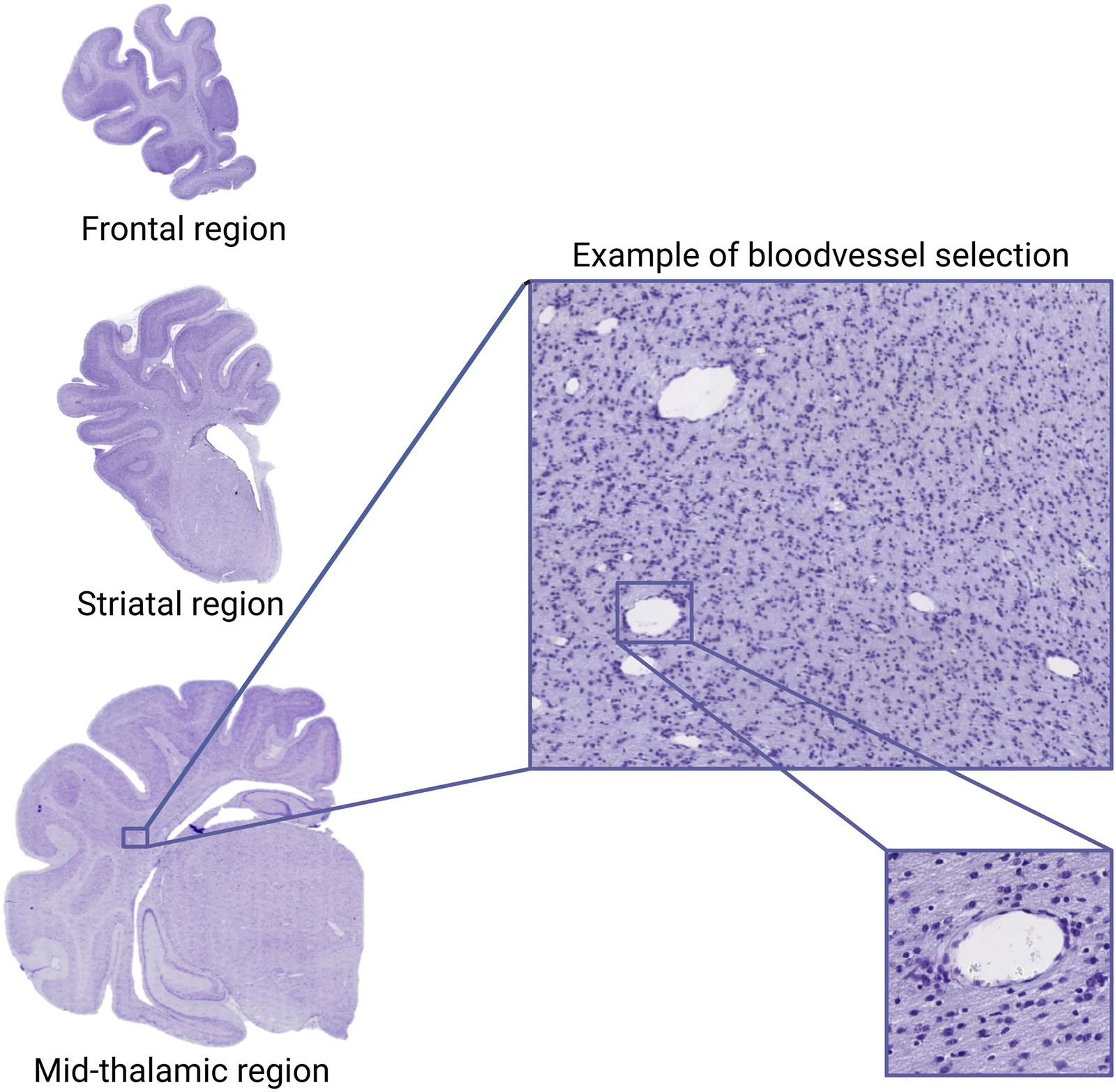
**Brain regions of interest analyzed through immunohistochemistry.** Nissl staining of the selected regions of interest for immunohistochemical staining of the paraffin-embedded coronal brain sections. Coronal sections are analyzed at the plane of the frontal cortex, striatum and mid-thalamic region, which includes the hippocampus and lateral ventricles. In all three regions of interest, blood vessels of the blood-brain barrier (BBB) were selected.

### 2.6 Microglia isolation from ovine brain

Tissue from the left frontal lobe as well as the left occipital lobe were used for isolation and stored temporarily in Hibernate®-E medium (Gibco, Life Technologies Europe BV, Bleiswijk, NL). After removing the meninges and accompanying vessels, white matter and cortex were separated, weighed (2.5 g per tube) and cut into fine pieces followed by mechanical trituration and enzymatic dissociation in 0.25% trypsin (Gibco, Life Technologies Europe BV, Bleiswijk, NL) and 30 μg/mL DNase I (Sigma-Aldrich, Zwijndrecht, NL). After mechanical dissociation and centrifugation the pellet was resuspended in 22% (vol/vol) Percoll (GE Healthcare, Sigma-Aldrich, Zwijndrecht, NL), 40 mM NaCl and 77% (vol/vol) myelin gradient buffer (5.6 mM NaH2PO4, 20 mM Na2HPO4, 140 mM NaCl, 5.4 mM KCl, 11 mM glucose, pH 7.4). Myelin was removed and the remaining pellet of mixed brain cells were resuspended in MACS buffer (0.5% BSA, 2 mM EDTA in PBS pH 7.2) and incubated with CD11b-FITC (Biorad, Puchheim, DE). Samples were subsequently washed and incubated with anti-FITC conjugated magnetic microbeads (Miltenyi Biotec B.V., Leiden, NL). Cells were washed and magnetically sorted according to the manufacturer's protocol (Miltenyi Biotec B.V., Leiden, NL). Labelled cells were counted and used for culture, or frozen in serum free freezing medium (PromoCell, Bio-Connect B.V., Huissen, NL). Primary microglia were cultured in high glucose DMEM supplemented with 5% heat-inactivated FBS and 1% Pen/Strep (Gibco, Life Technologies Europe BV, Bleiswijk, NL).

### 2.7 Flow cytometry

To assess purity and quality of the isolated primary microglia, 1.5 x 10^5^ cells/well in a 96-well plate were stained in PBS with a fixable viability stain 780 (BD Biosciences, Vianen, NL) followed by anti-CD45-biotin (Biorad, Puchheim, DE) coupled to a streptavidin V450 (BD Biosciences, Vianen, NL) in FACS buffer (5% FBS, 2 mM EDTA in PBS). Samples were fixed in PFA and acquired with a FACS Canto II (BD Biosciences, Vianen, NL). Live microglia were gated with a CD11b^+^ CD45intermedate (CD45^int^) profile (Galatro et al., 2017a, 2017b). Appropriate isotype and compensation controls were used for all conditions. 10.000 events were sampled for compensations and 5 x 10^4^ events were acquired for cell isolation.

### 2.8 Quantitative real-time polymerase chain reaction

Microglial inflammatory mediators were detected using the quantitative real-time polymerase chain reaction (qRT-PCR). Thereby, several immunomodulatory mediators were compared across the different treatment groups (Supplementary, Table 1). mRNA from cortex and white matter microglia was isolated separately using the RNeasy® Plus Micro Kit (QIAGEN Benelux B.V., Venlo, NL). Concentrations of RNA were measured using Nanodrop (Nanodrop Technologies, Llc, Wilmington, DE, US) followed by cDNA synthesis according to the manufacturer's protocol using the SensiFAST® cDNASynthesis Kit (QIAGEN Benelux B.V., Venlo, NL). Real-time PCR was performed using a 2.5 ng cDNA per reaction. Sensimix SYBR® Hi-Rox Kit (Bioline Reagents Limited, London, UK) was executed following the standard protocol provided by the manufacturer. Gene expressions were corrected with the reference genes HPRT 1, OvRPS 15 and YWHAZ. RNA yield was insufficient for qPCR analysis in three animals, resulting in a reduced sample size for the corresponding groups. Data is displayed as fold change to the control group (SAL-SAL).

**Table 1:** Animal characteristics.

	Sham-saline	Sham-MAPC	LPS-saline	LPS-MAPC
N (female, %)	5 (60)	5 (40)	6 (66,67)	5 (60)
Bodyweight (g)	3410 *(1845-3640)*	3000 *(2475-3378)*	2845 *(2537-3378)*	3660 *(3143-3972)*
Brain weight (g)	36.10 *(28.39-41.25)*	36.60 *(36.47-43.65)*	37.96 *(34.27-39.61)*	43.13 *(40.76-68.87)*
Brain-to-body weight (g/g)	1.28 *(0.86-1.92)*	1.21 *(1.17-1.74)*	1.24 *(1.13-1.44)*	1.29 *(1.09-1.94)*

Data presented as absolute number and percentages for sex, and data presented as median with interquartile range for brain- and bodyweight in grams (g). LPS = lipopolysaccharide, MAPC = Multipotent adult progenitor cells, g = grams.

### 2.9 RNA sequencing of cortical microglia

Two to four randomly selected animals per group were used for RNA bulk sequencing. Total RNA from cortical isolated microglia was extracted using the NucleoSpin RNA Midi Kit (Macherey-Nagel), following the manufacturer's protocol. Eluted RNA was subsequently concentrated to a final volume of 16 μL using the NucleoSpin RNA Clean-Up Kit (Macherey-Nagel). Library preparation and Illumina sequencing were performed at the Ecole normale supérieure GenomiqueENS core facility (Paris, France). Messenger (polyA+) RNAs were purified from 150 ng of total RNA using oligo(dT). Libraries were performed using the strand specific RNA-Seq library preparation *Stranded mRNA Prep, Ligation kit* (Illumina) and were multiplexed by 2 on a P3 flowcell. (Illumina). A 68 bp single or paired end read sequencing was performed on a NextSeq 2000 device (Illumina). A mean of 72.21 ± 9.10 million passing Illumina quality filter reads was obtained for each sample.

The analyses were performed using the Eoulsan pipeline (Jourdren et al., 2012) including read filtering, mapping, alignment filtering, read quantification, normalisation and differential analysis: before mapping, poly N read tails were trimmed, reads ≤40 bases were removed, and reads with quality mean ≤ 30 were discarded. Reads were then aligned against the *Ovis aries* genome from Ensembl version 112 using STAR (version 2.7.8a) (Dobin et al., 2013) Alignments from reads matching more than once on the reference genome were removed using Java version of samtools (Li et al., 2009). To compute gene expression, *Ovis aries* GTF genome annotation version 112 from Ensembl database was used. All overlapping regions between alignments and referenced exons:q1were counted and aggregated by genes using HTSeq-count 0.5.3 (Anders et al., 2015). The sample counts were normalized using the R Package DESeq2 1.8.1 (Love et al., 2014). Differential expression results were extracted via the Wald test, and p-values were adjusted using the Benjamini-Hochberg FDR method. Genes were considered significantly differentially expressed as FDR<0.01. Functional annotation was performed using g:Profiler (version *e113_eg59_p19_f6a03c19*) with g:SCS multiple testing correction method applying significance threshold of 0.05 (Kolberg et al., 2023). Additional graphs were made using the R package ggplot2 (Wickham, 2016). RNA sequencing raw data are deposited in the Gene Expression Omnibus (GEO) with accession number GSE283577.

### 2.10 Efferocytosis assay of microglia

To investigate whether *in vivo* primed microglia display different morphology or aberrant phagocytic capacity in response to restimulation with LPS (O55:B5; L2880, Sigma-Aldrich, Zwijndrecht, NL) *in vitro* an efferocytosis assay was performed. Therefore 5 x 10^5^ cells/well in a 96-well plate from a 3 animals per treatment, were stimulated after 3 days in culture with 100 ng/mL LPS, which is an established amount to induce pro-inflammatory cytokines (McArthur et al., 2010a) or treated with a vehicle (PBS). Two days later, Jurkat cells were labelled with 5, -6 Carboxyfluorescein diacetate succini-midyl ester (CFSE, Life Technologies Europe BV, Bleiswijk, NL). CFSE labelled Jurkat cells (1 × 10^6^ cells/ml) were incubated with 200 ng/mL anti-CD95 antibody (Beckman Coulter, Marseille Cedex, F) until at least 30% Jurkat cells were apoptotic (2-2.5h) as described previously (Schutters et al., 2013). Rate of apoptosis in CFSE labelled cells was monitored by a BD Accuri™ C6 Cytometer (BD Biosciences, Vianen, NL) for anti-ANXAV-AF647. An aliquot of 50 μl cell suspension was added to 220 μl binding buffer (10 mM HEPES/NaOH, pH 7.4, 150 mM NaCl, 5 mM KCl, 1 mM MgCl2 and 2.5 mM CaCl2) and 2.5 μl of ANXAV-AF647 (25 μg/mL) was added. Apoptotic CFSE labelled Jurkat cells were added to microglia in culture for efferocytosis in a ratio 3:1 (McArthur et al., 2010a) and incubated for 2 h based on previous results (see supplementary data). Microglia were subsequently fixed with 4% PFA. The plate was then analyzed with a Cytation3 microplate reader (BioTek Instruments, Bad Friedrichshall, DE). Six images were taken per well with 200× magnification. Images were processed with Image J software to overlay bright field and FITC channel and the efferocytotic capacity was calculated by the following equation:

$$percentage\;of\;phagocytising\;MG\left(\%\right)=\left(\frac{n\;of\;MG\;positive\;for\;apoptotic\;bodies\;}{total\;n\;of\;MG\;}\right)\times 100$$

$$efferocytotic\;capacity=\frac{\sum_{n=1}^{\infty }amount\;MG\;n\;apoptotic\;body}{total\;amount\;of\;MG\;}$$

Internalization of apoptotic bodies by microglia was confirmed by confocal microscopy at 600× magnification (Leica DMI 4000, Leica Microsystems; Fig. 3).

**Fig. 3:**
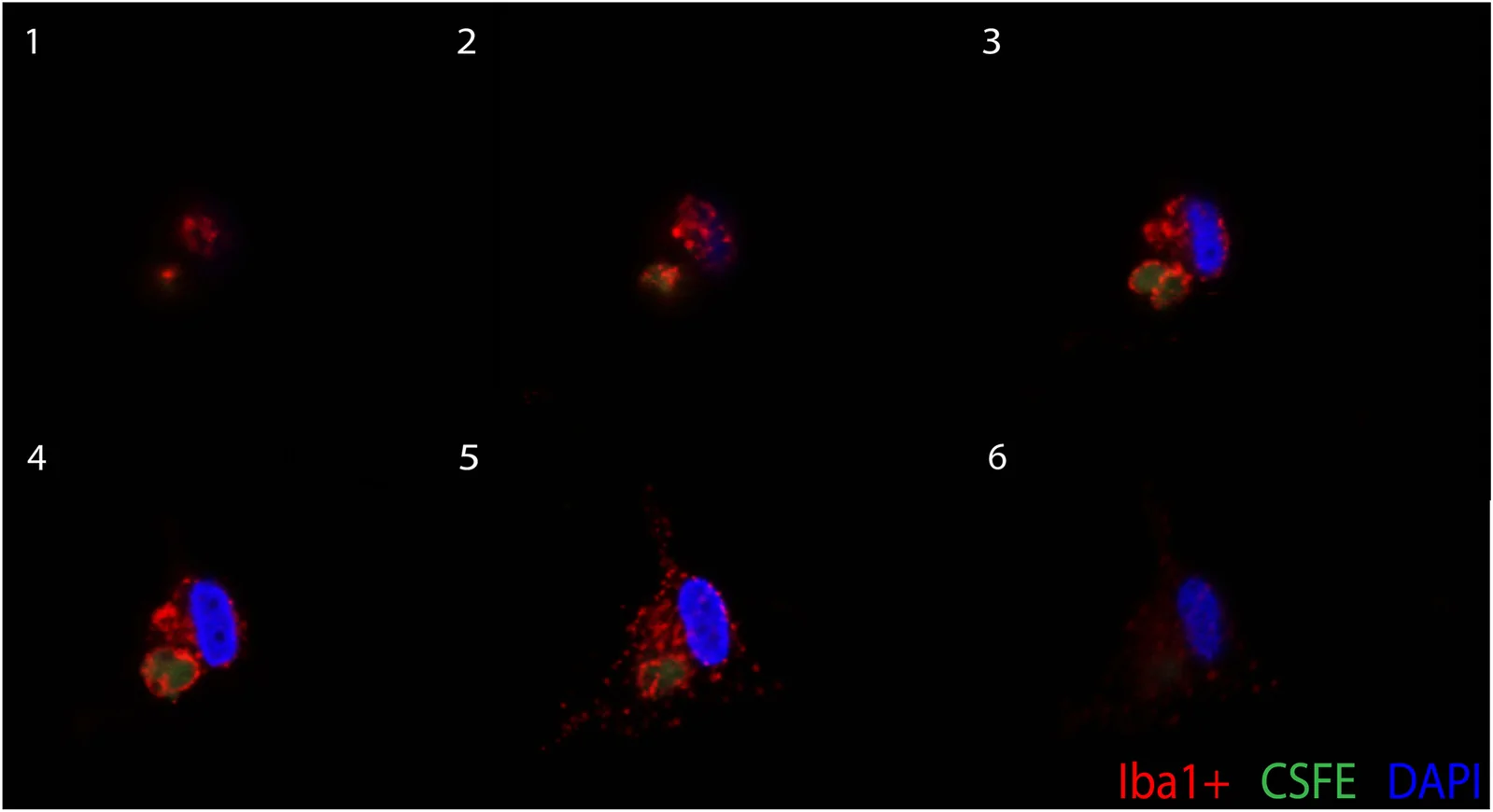
**Representative images of apoptotic body by cortical microglia.** Z-stack of different focal planes as example of apoptotic body (CSFE) uptake by cortical microglia (IBA-1+) over time, verified by confocal microscopy.

### 2.11 Immunohistochemistry of paraffin embedded sections

4 μm sections from each brain region were cut with a microtome (Leica RM2235, Leica Microsystems, Wetzlar, Germany). Immunohistological staining was performed on mainly adjacent slides within the regions of interest of each fetal brain. Peripheral immune cells and immune cells within the brain parenchyma were assessed by staining for general immune cell antigen (CD45; 1:1000, monoclonal mouse IgG1 anti-sheep CD45, MCA2220GA, Bio-Rad) and ANXA1 (1:300, polyclonal rabbit anti-ANXA1, ab137745, Abcam). Ionized calcium-binding adaptor protein (IBA-1; 1:1000, polyclonal rabbit anti-IBA-1, 019-19741, Wako Pure Chemical Industries, Osaka, Japan) expressed in round cells within the circulation was used as a marker for differentiating monocytes (Jeong et al., 2013). The quantity of circulating neutrophils was assessed by the immunohistochemical marker myeloperoxidase (MPO; 1:200, polyclonal rabbit anti-human MPO, A0398, DAKO Agilent Technologies, Santa Clara, CA) (Jeong et al., 2013). Deparaffinization and rehydration was performed by incubation in xylol and decreasing alcohol concentrations. Endogenous peroxidase activity was blocked by incubation in 0.3% H_2_O_2_ diluted in phosphate buffered saline (pH 7.4; CD45, IBA-1, MPO) or 0.3% H_2_O_2_ diluted in TBS (pH 7.4; ANXA1) for 20 min. Antigen retrieval for CD45, IBA-1 and ANXA1 involved boiling tissues in 10 mM citrate buffer (pH 6.0) for a total of 10 min. Non-specific binding was prevented by incubation with 4% normal goat serum for 1 h. Tissues were incubated with the primary antibody in a humidified box at 4°C overnight. MPO was incubated with PBS supplemented with 0.1% Triton X-100. Overnight incubation was followed with the secondary antibody at 1:200 (polyclonal goat anti-mouse biotin, E0433, DAKO (CD45), polyclonal swine anti-rabbit biotin, E0353, DAKO (IBA-1, ANXA1) or 1:50 (polyclonal goat anti-rabbit HRP, 111-035-045, Jackson ImmunoResearch, West Grove, PA (MPO)) for 1 h at room temperature. For CD45, IBA-1 and ANXA1 the antigen-specific signal was enhanced with a Vectastain ABC peroxidase Elite kit (Vector Laboratories Inc., Burlingame, CA) for 1 h and 3,3′-diaminobenzide (DAB) for 1-4 min. Tissues were incubated with Mayer's haematoxylin followed by increasing dehydration alcohol concentrations and xylol. Researchers performing the analyses were blinded for the experimental conditions. Paraffin embedded slides were scanned using a light microscope (Ventana iScan, Ventana Medical System, Inc., Tucson, AZ). Positive cells were counted and expressed as total cell count per square millimeter (cells/mm^2^), averaged per region of interest. Analyses were performed using QuPath v0.2.0-m8 [41]. (Co-)stained cells were counted manually and expressed per tissue area (mm^2^) measured by QuPath v0.4.3 (Bankhead et al., 2017).

### 2.12 Immunohistochemistry of isolated microglia

Purity of primary microglia isolation was confirmed by immunohistochemistry. 5 x 10^4^ cells/well in a dark 96-well plate were fixed with 4% PFA for 10 min followed by incubation of 1h at RT with IBA-1 (1:500, Wako Pure Chemical Industries, Osaka, Japan). The secondary antibody goat anti-rabbit TXRED (1:500, Southern Biotech, Birmingham, AL, US) was added for 1h at RT and afterward cell nuclei were counterstained with DAPI. Cells were left in PBS until imaging with Cytation3 microplate reader (BioTek Instruments, Bad Friedrichshall, DE).

### 2.13 Statistical analysis

Statistical analyses were performed in GraphPad Prism (version 10.6.1; GraphPad; San Diego, CA). All values were computed per animal as mean and standard deviation. Statistical differences were confirmed by performing a two-way ANOVA with a Turkey's multiple comparisons test. Statistical significance was set at alpha = 0.05. P values < 0.1 were considered an effect with possible biological relevance (Willems et al., 2016; Gussenhoven et al., 2017).

## 3 Results

### 3.1 Characteristics of instrumented ovine fetuses

Animal characteristics are shown in Table 1. There were no significant differences between experimental groups when comparing sex, bodyweight, brain weight or brain-to-body weight.

### 3.2 Isolation method yields microglia from white and grey matter with high purity

The isolation method used resulted in pure microglia from both white matter and cortex (Fig. 4). Isolated cells displayed typical microglial morphology that stained positive for microglia marker IBA-1 (Fig. 4A). Flow cytometric analysis revealed highly viable cells and microglia purity >99% of all gated live cells (Fig. 4B). The applied gating strategy, together with the specific marker profile, indicates the absence of significant contamination by other cell types.

**Fig. 4:**
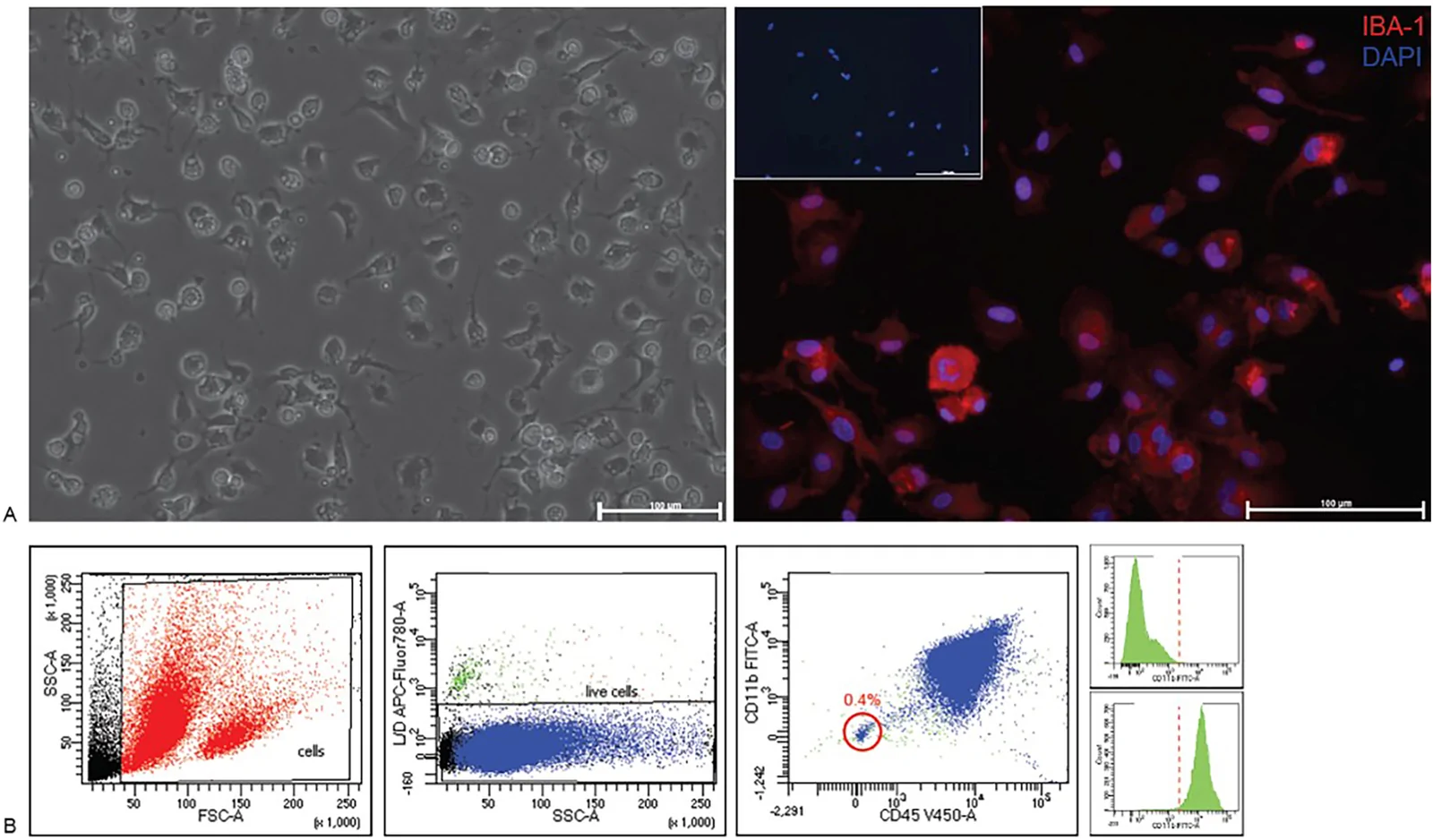
**Isolation of pure fetal primary microglia.** A) Representative image of typical morphology after three days *in vitro* culture (left panel, 100× magnification) and immunohistochemistry of primary microglia for microglia marker IBA-1 (right panel, 200× magnification). B) Representative quality check by flow cytometry and gating strategy for microglia. Total cells of freshly MACS sorted cells (left), positive selection of live cells for negative signal of live dead (L/D) marker FVS780 (second panel), CD11b + -FITC labelled microglia stained for CD45biotin-strepV450 (third panel), histogram of FITC signal from cells washed though MACS column (flow through, CD11b-cells, upper histogram) and cells eluted from column after removal out of magnetic field (CD11b + cells, lower histogram).

### 3.3 Grey matter sorted microglia have differential gene expression profiles after LPS exposure and MAPC treatment

To investigate the molecular mechanism underlying the microglia response to prenatal inflammation and subsequent MAPC therapy, bulk RNA sequencing was conducted on cortical microglia isolated from the four different experimental groups.

Differential gene expression analysis between preterm birth with and without LPS exposure identified 565 differently expressed genes (DEGs, Fig. 5A). Stem cell administration in saline conditions revealed 298 DEGs compared to saline alone (Fig. 5B). MAPC therapy after intra-amniotic LPS exposure provoked a difference of 3124 DEG compared to the LPS-saline group (Fig. 5A). When comparing experimental groups with MAPC therapy with or without LPS exposure, 298 DEGs were identified (Fig. 5A).

**Fig. 5:**
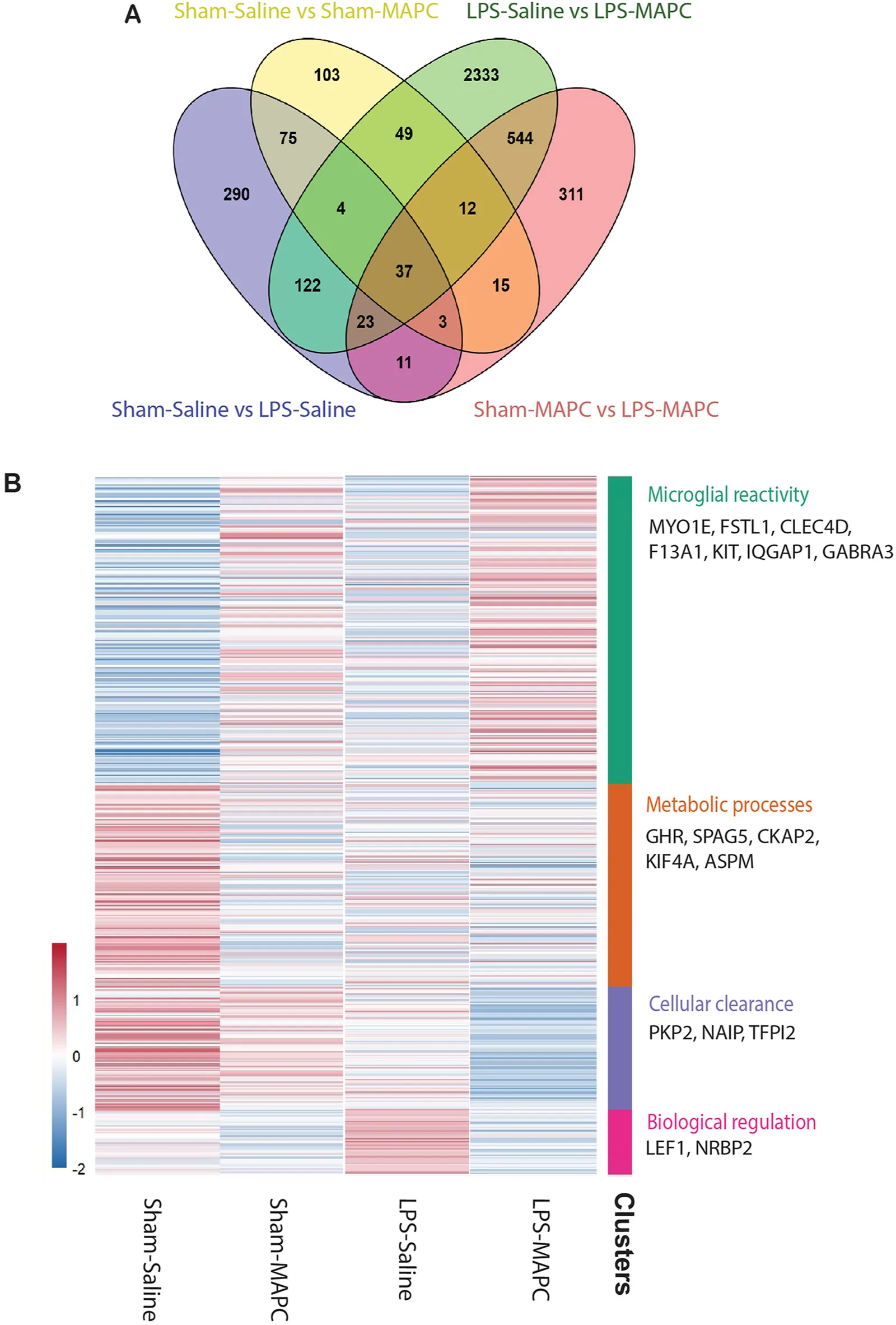
**RNA-sequencing of cortical microglia.** Venn's diagram of the four comparisons (A). Heatmap of the mean normalized gene count per experimental group with per cluster the GO terms and relevant genes (B). Group sizes were as follows: Sham-Saline (n = 2), Sham-MAPC (n = 4), LPS-Saline (n = 4) and LPS-MAPC (n = 4). LPS = lipopolysaccharide, SAL = saline, MAPC = Multipotent adult progenitor cells.

Heatmap analysis of the mean normalized gene count revealed four distinct clusters: microglial reactivity, metabolic processes, cellular clearance and biological regulation (Fig. 6B). Within these clusters relevant genes were identified by the analysis of the 20 most significant genes for each group comparisons (Sham-saline vs LPS-saline, Sham-saline vs Sham-MAPC, LPS-saline vs LPS-MAPC and Sham-MAPC vs LPS-MAPC) (Supplementary Fig. 1). Intra-amniotic LPS-exposure led to an upregulation of genes associated with microglial activation after brain damage (GHR, SPAG5, CKAP2) and downregulation of genes required to exert the immunomodulatory function in microglia (MYO1E, FSTL1, LEF1). Neuroinflammation-associated genes which were upregulated after LPS exposure, were downregulated after MAPC treatment in inflammatory conditions (CLEC4D, F13A1, KIT). MAPC therapy also induced an increase in genes associated with microglial phagocytosis (NRBP2), mitophagy (PKP2) and downregulation of IQGAP1 which is associated with leukocyte trafficking across peripheral endothelium. Compared to MAPC therapy in sham conditions, microglia from LPS-MAPC animals showed an upregulation of neuroprotective genes (NAIP, TFPI2). Interestingly, intra-amniotic LPS exposure downregulated GABRA3, known to be dysregulated after preterm birth and involved in ADHD and autism. LPS exposure upregulated genes which are associated with intellectual disabilities and developmental delays (KIF4A, ASPM) which was reversed after MAPC treatment.

**Fig. 6:**
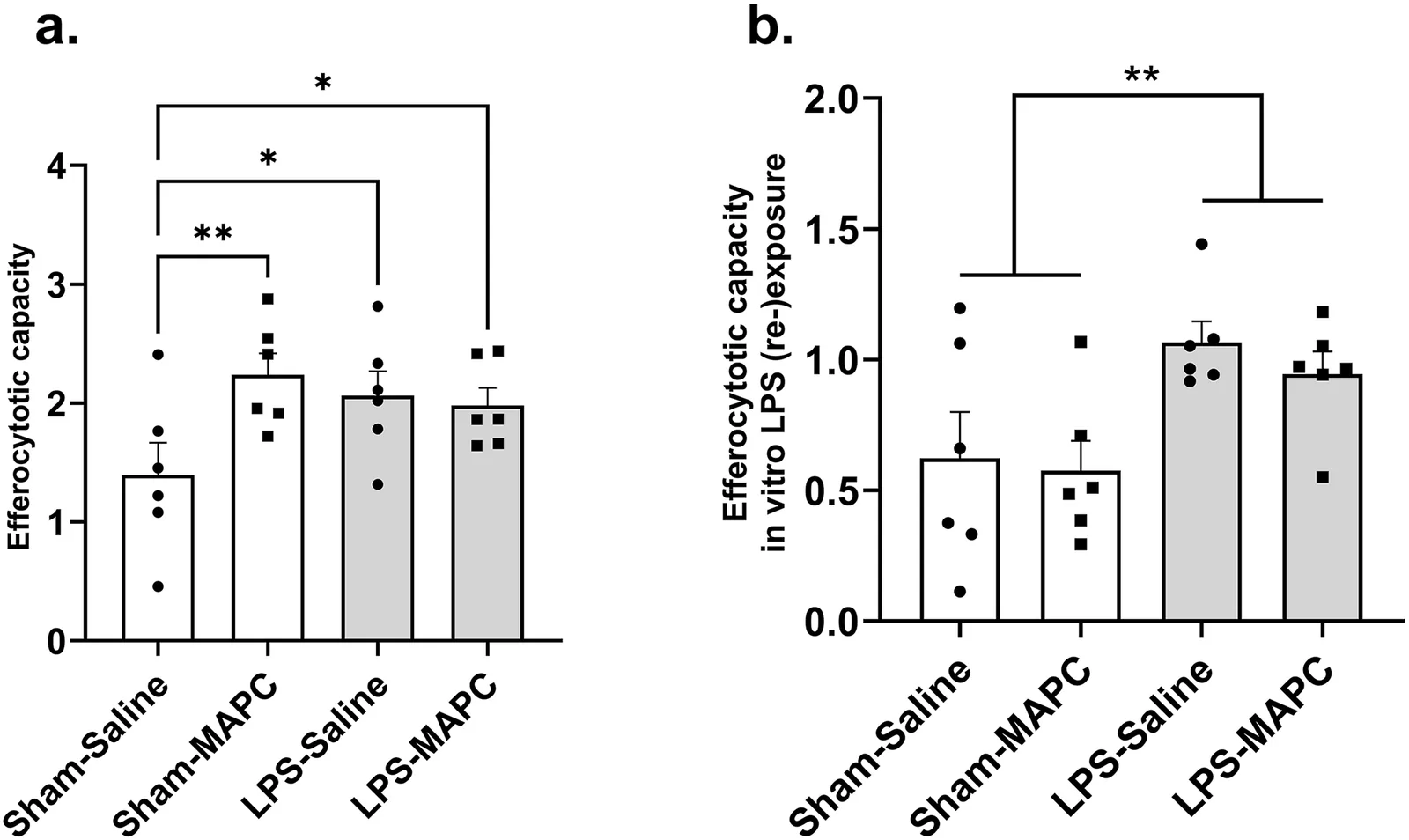
**Efferocytotic capacity of cortical microglia (a.) and after *in vitro* LPS (re-)exposure (b.).** Summary of the two-way ANOVA results are presented on each panel. Data are shown as mean ± SEM. Two biological replicates per group (n = 2) were used, with each sample assayed in triplicate, as shown in the graph. Results of the post-test are indicated with: *p < 0.05, **p < 0.01. LPS = lipopolysaccharide, SAL = saline, MAPC = Multipotent adult progenitor cells.

### 3.4 Microglia are primed by intra-amniotic LPS exposure and after MAPC therapy efferocytotic capacity of microglia was preserved

LPS exposure and/or MAPC therapy increased efferocytotic capacity in isolated cortical microglia compared to cortical microglia isolated from Sham-saline animals (p = 0,003 for sham-MAPC, p = 0.017 for LPS-saline and p = 0.039 for LPS-MAPC, Fig. 6A). After *in vitro* (re-)exposure to LPS, a significant difference was found between the sham groups (sham-saline and sham-MAPC) and the LPS groups (LPS-saline and LPS-MAPC, p = 0.003), indicative for LPS priming and a retained efferocytotic capacity upon MAPC treatment after LPS exposure *in vitro* (Fig. 6B).

### 3.5 MAPC therapy in the initial phase of inflammation, resulted in an accumulation of neutrophils and leukocytes expressing the proresolving mediator ANXA1

At the BBB, neutrophils (MPO+) and monocytes (IBA-1+) were significantly increased in LPS-MAPC animals compared to untreated animals exposed to LPS (p = 0.006 for MPO+, p = 0.023 for IBA-1+, Fig. 7A). Adjacent staining of leukocytes (CD45^+^) and proresolving mediator ANXA1, revealed a significant increase of immunomodulatory immune cells in LPS-MAPC animals compared to untreated animals exposed to LPS, at the BBB (p = 0.007 for CD45^+^, p = 0.075 for ANXA1+, Fig. 7A) and in brain parenchyma in (p = 0.006 for CD45^+^, p = 0.015 for ANXA1+, Fig. 7B). However, no significant changes in the number of microglia (IBA-1+) in the brain parenchyma was found between the experimental groups (Fig. 7B). Due to their essential role in mediating brain injury by transducing systemic inflammation into neuroinflammation, we further investigated the microglial expression profile.

**Fig. 7:**
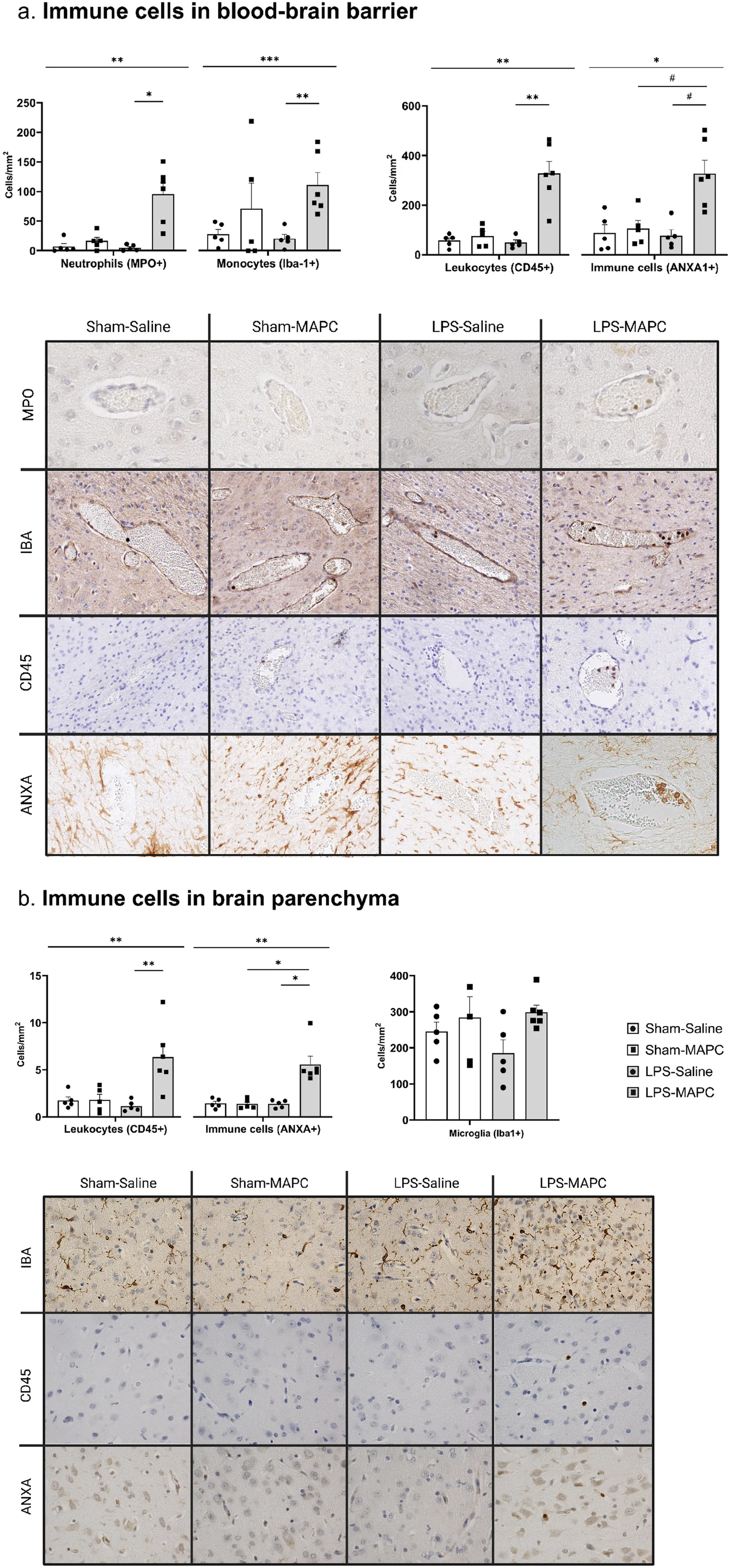
**Leukocytes at blood-brain barrier (A.) and within white matter (B.).** Data are shown as mean ± SEM. Group sizes were as follows: Sham-Saline (n = 5), Sham-MAPC (n = 5), LPS-Saline (n = 5) and LPS-MAPC (n = 6). Summary of 2-way ANOVA results are presented on each panel with p-values represented as #p < 0.1, *p < 0.05 and ***p < 0.0001. LPS = lipopolysaccharide, SAL = saline, MAPC = Multipotent adult progenitor cells.

### 3.6 Isolated microglia from white and grey matter exhibit increased pro-and anti-inflammatory expression profile after intra-amniotic LPS exposure followed by MAPC treatment

Intra-amniotic LPS exposure and MAPC therapy resulted in upregulation of mRNA expression of pro- and anti-inflammatory mediators in microglia from white matter and cortex compared to all other treatment groups (Fig. 8). Changes in anti-inflammatory cytokine expression were only observed in cortical microglia. ANXA-1, IL-10 and galectin mRNA expression levels were significantly increased in animals treated with MAPC after antenatal LPS, compared to sham-MAPC and LPS-Saline animals (respectively p = 0.019 and p = 0.016 for ANXA1, p = 0.038 and p = 0.003 for IL10, p = 0.0008 and p=<0.0001 for galectin). Pro-inflammatory cytokines were significantly higher expressed in microglia from white matter and cortex in the LPS-MAPC animals compared to sham-MAPC and LPS-saline experimental groups (respectively for white matter p = 0.032 and p = 0.009, for cortex p = 0.01 and p = 0.0003 for IL-8 and p=<0.0001 for all significant comparisons for COX-2). Immune mediators CD68, Socs3, CD206, CD209, IL-1β and TNFα are shown, in Supplementary Fig. 2 with no significant differences observed between experimental groups for SOCS3, CD209 and TNFα. CD68 expression was elevated in cortical microglia from animals in the LPS-MAPC group compared to cortical microglia from untreated animals exposed to intra-amniotic LPS (p = 0.013). MAPC treatment after LPS exposure led to an increase in the pro-inflammatory cytokine IL-1 β compared to untreated animals exposed to LPS (p = 0.014). In LPS-MAPC animals CD206 mRNA levels were significantly increased compared to sham-MAPC animals (p = 0.042, Supplementary Fig. 2).

**Fig. 8:**
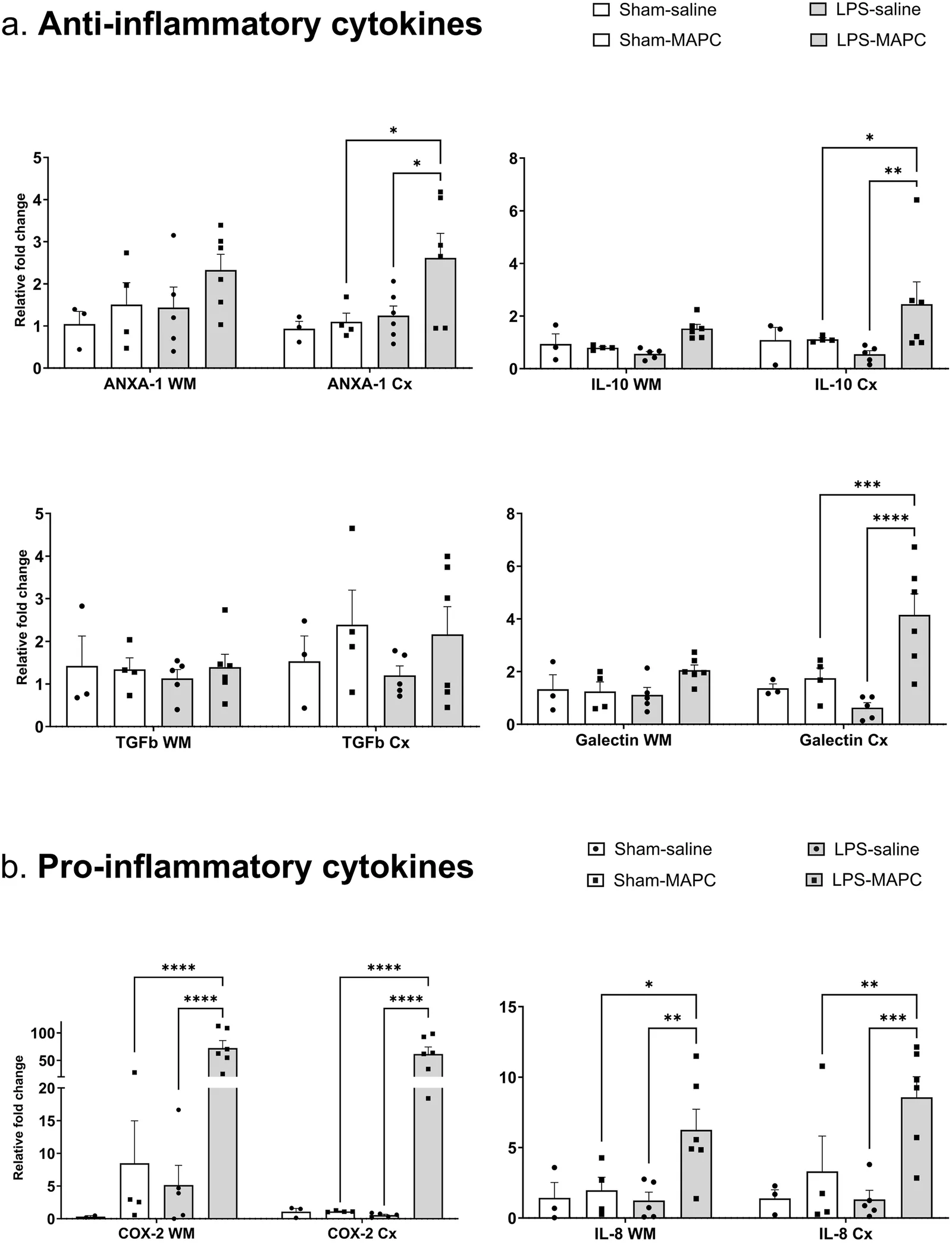
**mRNA expression of anti-inflammatory (a.) and pro-inflammatory (b.) mediators in white matter (WM) and cortex (Cx) microglia.** Data are shown as mean ± SEM. Group sizes were as follows: Sham-Saline (n = 3), Sham-MAPC (n = 4), LPS-Saline (n = 5) and LPS-MAPC (n = 6). Summary of the ANOVA results are presented on each panel. Results of the post-test are indicated with: *p < 0.05, **p < 0.01, ***p < 0.001, ****p < 0.0001. LPS = lipopolysaccharide, SAL = saline, MAPC = Multipotent adult progenitor cells.

## 4 Discussion

Preterm birth, often mediated by systemic inflammation, affects neurodevelopment, causing long-lasting cognitive and behavioral impairments (Fleiss et al., 2021; Paton et al., 2017b). Crosstalk of peripheral immune mediators over the blood-brain barrier can activate neuroinflammation (Klein et al., 2022; Mottahedin et al., 2017). Microglia are essential in initiating and maintaining neuroinflammation making them an important therapeutic target (Fleiss et al., 2021). We show, in a preclinical ovine model for chorioamnionitis, that early administration of multipotent adult progenitor cells (MAPC), alters the neuroinflammatory trajectory by modifying microglial phenotype and transcriptome towards immunomodulation possibly through the accumulation of pro-resolving peripheral immune cells to BBB.

In this ovine model, chorioamnionitis was established by intra-amniotic LPS administration resulting in fetal immune activation indicated by elevated systemic IL-6 and multiorgan inflammation, including the brain (Klein et al., 2022; Neuen et al., 2024; Hoogenboom et al., 2022; van Gorp et al., 2021). In this study, LPS exposure primed peripheral immune cells at the blood-brain barrier. Moreover, intra-amniotic LPS exposure affected microglial function and its transcriptome. Notably, at this timepoint, LPS did not induce overt neuroinflammation, a finding potentially attributable to the timing of LPS administration and tissue analysis, as we have previously demonstrated (Gussenhoven et al., 2018). Despite the absence of robust neuroinflammatory markers at the studied timepoint, transcriptomic differences were detected between sham- and LPS-treated animals, and MAPC treatment produced distinct immunomodulatory effects in LPS-exposed animals compared with controls. These data suggest that underlying biological reprogramming occurs following intrauterine inflammation that may not be fully captured by conventional outcome measurements, highlighting the importance of molecular profiling in detecting early immune system perturbations and MAPC-induced immunoregulation.

This reprogramming creates a stimulating environment for stem cells to accomplish their immunomodulatory function, indicated by increased CD206 expression levels of microglia after MAPC therapy but only after LPS exposure. To further elucidate the trajectory in which MAPC therapy affects neuroinflammation, innate immune cells at the blood-brain barrier and in the brain parenchyma were characterized. Previously we showed in our preclinical model for chorioamnionitis that MAPC therapy elicits a coordinated anti-inflammatory response in the peripheral immune system, potentially supporting improved neurological outcomes (Klein et al., 2022). In this study we observed an accumulation of neutrophils and monocytes at the blood-brain barrier in animals treated with MAPCs after antenatal infection. Additionally, immune cells colocalized with ANXA1, a key mediator to resolve inflammation (Perretti and D'Acquisto, 2009). Emerging evidence argues that ANXA1 could be transported towards injured tissues by neutrophils, followed by increased expression of ANXA1 in infiltrating macrophages causing anti-inflammatory polarization (McArthur et al., 2020). Through increased ANXA1 expression in innate immune cells after antenatal inflammation, MAPC therapy could possibly modulate the peripheral inflammatory response towards resolving neuroinflammation through microglial activation (McArthur et al., 2010b, 2020; Luo et al., 2014; Ries et al., 2016; Loiola et al., 2019). This effect of MAPC therapy is solely found in animals with antenatal inflammation, which could be explained by the fact that adult progenitor mesenchymal stromal cells respond to inflammatory factors when preconditioned or primed with IL-6 or IL-8, following LPS exposure (Gussenhoven et al., 2017, 2018; Song et al., 2020; Ferreira et al., 2018; Saparov et al., 2016; Üçal et al., 2021; Domenis et al., 2018), which is important for monocyte recruitment and release extracellular vesicles which can drive macrophages and possibly adaptive immune cells like T-cells towards an anti-inflammatory phenotype.

Microglia are essential mediators of neuroinflammation by receiving peripheral inflammatory signals over the blood-brain barrier (Fleiss et al., 2021). Characterizing the immune cells in the brain parenchyma showed no changes in IBA-1 positive microglia between experimental groups, however, timely intervention of MAPC therapy after antenatal inflammation, induced an increase in both pro- and anti-inflammatory mediators. Notably, interleukin-10 (IL-10) in white matter and cortical microglia was increased in LPS-exposed animals treated with MAPC therapy. In the absence of MAPC therapy, LPS induces a decrease in IL-10in microglia from white matter regions in almost all animals. Accumulating literature reports the vital role of IL-10 in preventing excessive and damaging inflammatory processes due to its anti-inflammatory capacities (Lobo-Silva et al., 2016; Dammann and O'Shea, 2008). The absence of IL-10 abolishes M2 polarization (Laffer et al., 2019), which is essential for re-establishing brain homeostasis after injury and highlighted by the observation that the endogenous ability to produce IL-10 is associated with lower incidences of white matter abnormalities in preterm infants (Dammann and O'Shea, 2008; Dördelmann et al., 2006). In addition, stem cell treatment has been shown to result in elevated systemic IL-10 levels and induced IL-10 expression in monocytes/macrophages (Yang et al., 2017; Wang et al., 2019; de Witte et al., 2018; Walker et al., 2010). The MAPC-driven induction of IL-10 may be mechanistically explained by the elevated production of ANXA1 by microglia, as demonstrated in our study. ANXA1 induces IL-10 production in microglia or macrophages and promotes phagocytosis (Jeong et al., 2013; McArthur et al., 2010b; Ries et al., 2016; Loiola et al., 2019; Ferlazzo et al., 2003; Guido et al., 2013; Cooray et al., 2013). The association between ANXA1 and IL-10 is strengthened by *in vitro* and *in vivo* findings, which show that exogenous administration of ANXA1 in IL-10 deficient mice fails to prevent tissue injury in an intestinal ischemia/reperfusion model, and isolated macrophages from ANXA1 knockout mice, exhibit a blunted IL-10 response (Souza et al., 2007; Qin et al., 2017). Furthermore, we report an increase in pro-inflammatory cytokines such as COX-2, in microglia of animals treated with MAPC therapy after antenatal infection. COX-2 is key mediator in the inflammatory process, synthesizing the first step of prostaglandin production which is needed for vasodilatation (Choi et al., 2009). Although COX-2 is predominantly cited as pro-inflammatory marker of microglia, it has been shown that COX-2 is needed for anti-inflammatory polarization in developmental processes (Na et al., 2013; Ho et al., 2011). Therefore, the data suggests that MAPCs orchestrate neuroinflammation through both pro- and anti-inflammatory mediators, this supporting our hypothesis that MAPCs prevent persistent neuroinflammation by directing microglia towards a more favorable immunological landscape (Meng et al., 2024). However, the precise mechanism underlying this dual response, its implications for brain function and the therapeutic potential of MAPCs based interventions remain to be elucidated. Further investigation is needed to characterize the underlying immune response pathways in this context.

The efferocytotic capacity increased following MAPC treatment in sham animals, suggesting a direct immunomodulatory effect on microglia independent of inflammatory priming. This finding suggests that MAPCs may drive microglia toward a more homeostatic or surveillance-like phenotype, characterized by increased apoptotic cell clearance. Since efficient removal of apoptotic cells and cellular debris is critical for brain development and tissue homeostasis (Silva et al., 2024), modest increases in efferocytosis under non-inflammatory conditions may be beneficial. Conversely, excessive phagocytic activity in the developing brain could potentially disrupt synaptic pruning (Dundee et al., 2023). Although we observed no adverse effects in the current study, these results underscore the need for comprehensive investigation of MAPC effects in non-injured models.

Transcriptomic analysis of microglia revealed distinct transcriptional profiles, indicating that prenatal inflammation and stem cell therapy modulate microglial gene expression towards immunomodulation. Differential gene expression analysis identified significant changes, particularly in pathways related to efferocytosis, cellular senescence, and phagocytosis, which were enriched in both LPS-exposed and stem cell-treated groups. A previous *in vitro* study of LPS-exposed microglia showed similar changes, with DEGs linked to proliferation, phagocytosis and the release of pro-inflammatory cytokines (Pulido-Salgado et al., 2018). When MAPC therapy was combined with LPS exposure, a more pronounced transcriptional response was observed, with over 3000 DEGs and additional enrichment in pathways like microglial reactivity and cellular clearance. These results indicate that while MAPC therapy alone modulates microglial function, its impact is amplified in the context of prenatal inflammation, suggesting a complex interaction between inflammation and therapeutic intervention in priming microglial responses. Similar transcriptomic findings are reported in a mouse model of neuroinflammation where distinct subtypes of activated microglia were revealed in response to perinatal brain injury, induced by IL-1 β (Dufour et al., 2025). The identification of microglial subsets with unique transcriptional profiles opens new research questions how these different microglial subsets are affected by inflammation and stem cell treatment over time.

Next to the immunomodulatory effect of MAPC therapy on the microglial transcriptome, we identified upregulation of genes associated with developmental delays and neurocognitive disabilities following intra-amniotic LPS exposure, after intra-amniotic LPS exposure, which was partly reversed by MAPC treatment during the initial phase of neuroinflammation. The long-term neurodevelopmental impairments described with perinatal brain injury, develop over time through cumulative effects of multiple perinatal insults disrupting ongoing cerebral maturation (Yates et al., 2021). This study demonstrated genomic alterations at a very early stage of neuroinflammation a developmentally vulnerable period characterized by rapid brain maturation when the fetal brain is particularly susceptible to inflammatory injury and transcriptomic changes may precede overt structural damage (Chen et al., 2022; Oldenburg et al., 2020). These early transcriptomic signatures associated with neurodevelopmental delays reveal the vulnerability of the developing brain during late gestation and underscore the therapeutic potential of stem cell interventions when initiated in the early inflammatory phase, before cascading molecular and cellular changes establish persistent neurodevelopmental impairments (Chen et al., 2020; Oldenburg et al., 2020).

MAPCs have emerged as compelling therapeutic approach for central nervous system injury because of their robust immunomodulatory and regenerative properties (Ravanidis et al., 2017). In stroke specifically, MAPC therapy showed significant promise by attenuating post-injury inflammation and promoting tissue repair through modulation of systemic immune responses, particularly via splenic activation and altered macrophage polarization (Yang et al., 2017). The clinical translation of allogeneic MAPC therapy marked a major milestone, as early clinical feasibility studies demonstrated the safety and viability of administering these cells to patients with brain injury (Jellema et al., 2015), with the allogeneic approach offering distinct advantages, most notably the ability to manufacture standardized, off-the-shelf cell product without the timing constraints that autologous approaches impose, making early intervention feasible especially in neonatal populations where rapid treatment is critical (Razak et al., 2025). This research is constrained by several important limitations that should inform interpretation of findings and guide future investigations.

A limitation of this study is the single timepoint of analysis, which may have overlooked both immediate inflammatory reactions and the temporal dynamics of neuroinflammatory progression. In the context of neuroinflammation following prenatal LPS exposure, biphasic systemic inflammatory responses have been documented in fetal models (Gussenhoven et al., 2018), with distinct temporal phases of cytokine elevation, resolution, and potential delayed escalation. This study provides a focused view of what is fundamentally a dynamic, evolving process. Consequently, this approach may have missed critical windows of acute immune activation or the longer-term persistence of microglial changes that characterize the neuroinflammatory response to perinatal insults. A second limitation involves restriction of brain tissue sampling to a single region, which introduces variability related to regional heterogeneity in neuroinflammatory responses and sampling procedures. Although the selected area is known to be particularly vulnerable in neonatal brain injury (Strahle et al., 2019), other regions including the thalamus and internal capsule may exhibit distinct inflammatory responses. Future studies should therefore include multiple brain regions and multiple timepoints to enable a comprehensive, spatiotemporal evaluation of neuroinflammatory and neurodevelopmental changes following perinatal inflammatory insults. Such approaches would better capture the complexity of regional heterogeneity and temporal dynamics inherent to perinatal brain injury pathophysiology.

Altogether, our findings suggest that administration of MAPC therapy in the initial stage of antenatal inflammation promotes an immunomodulatory state in microglia, characterized by both pro- and anti-inflammatory features together with enhancing immune crosstalk at blood-brain barriers through peripheral immune cells expressing the inflammation-resolving molecule ANXA1. We conclude that early intervention in the neuroinflammatory cascade by MAPC therapy directs the neuro-immune response while preserving microglial function. Such a coordinated and balanced inflammatory response is a prerequisite for resolving persistent neuroinflammation in perinatal brain injury (Fleiss and Gressens, 2012). Early intervention in the inflammatory cascade, targeting key inflammatory mediators could protect the brain from persistent neuroinflammation and prevent long-term developmental impairments (Haynes et al., 2013).

## CRediT authorship contribution statement

**Valéry L.E. van Bruggen:** Writing – review & editing, Writing – original draft, Visualization, Methodology, Investigation, Formal analysis, Data curation, Conceptualization. **Sanne J.C.M. Claassen:** Writing – review & editing, Writing – original draft, Visualization, Software, Methodology, Investigation, Formal analysis. **Luise Klein:** Software, Methodology, Formal analysis, Data curation, Conceptualization. **Cindy Bokobza:** Writing – review & editing, Validation, Software, Investigation, Formal analysis. **Jennifer Hua:** Software, Investigation, Data curation. **Tiphaine Marvillet:** Software, Investigation, Formal analysis. **Michelle M.E. Zandbergen:** Investigation, Formal analysis. **Charlotte van Gorp:** Writing – review & editing, Investigation, Formal analysis, Data curation. **Florian Caimant:** Writing – review & editing, Validation, Supervision, Software, Methodology. **Renate G. van der Molen:** Writing – review & editing, Supervision, Resources, Project administration, Funding acquisition. **Pierre Gressens:** Writing – review & editing, Supervision, Software, Resources, Project administration, Funding acquisition. **Daan R.M.G. Ophelders:** Writing – review & editing, Validation, Supervision, Software, Resources, Methodology, Conceptualization. **Tim G.A.M. Wolfs:** Writing – review & editing, Writing – original draft, Supervision, Resources, Project administration, Methodology, Funding acquisition, Data curation, Conceptualization.

## Ethics declaration

This study was conducted in accordance with the ARRIVE (Animal Research: Reporting of In Vivo Experiments) guidelines. This study was approved by the Central Authority for Scientific Procedures on Animals. (Approval No. AVD107002016784)

## Funding

Multipotent adult progenitor cells were supplied by Athersys Inc. (Cleveland, Ohio, USA)/Healios K.K. (Tokyo, Japan). The company was not involved in the study design, data collection, analysis, interpretation of results or preparation of the manuscript.

This work was supported by grants from Inserm, Université Paris Cité, Horizon 2020 Framework Program of the European Union (grant agreement no. 874721/PREMSTEM), Fondation Princesse Grace de Monaco; and an additional grant from “Investissement d'Avenir -ANR-11-INBS-0011-“NeurATRIS.

## Declaration of competing interest

The authors declare that they have no known competing financial interests or personal relationships that could have appeared to influence the work reported in this paper.

## Data Availability

Data will be made available on request.
