# Supplementary material for: Stem cells redirect the neuroinflammatory trajectory of primed microglia in an ovine chorioamnionitis model
Source: Brain Behav Immun Health. 2026 Sep 18;57:101359. doi: 10.1016/j.bbih.2026.101359 (PMC13634120; doi:10.1016/j.bbih.2026.101359)
Supplement: Supplementary file 1 [file mmc1.docx]

**Supplementary data**

**Supplementary table 1: Primers for qRT-PCR, fw= forward, rv=reverse.**

| Marker | Gene | | Sequence |
| --- | --- | --- | --- |
| Pro-inflammatory | COX-2 | fw | TAACACGCTCTACCACTGGCA |
|  |  | rv | GATTCCTACGACCAGCGACC |
|  | IL-8 | fw | GTTCCAAGCTGGCTGTTGCT |
|  |  | rv | GTGGAAAGGTGTGGAATGTGTTT |
|  | IL-1 β | fw | CGTCTTCCTGGGACGTTTTAG |
|  |  | rv | CTGCGTATGGCTTCTTTAGGG |
|  | TNF- α | fw | GCCGGAATACCTGGACTATGC |
|  |  | rv | CAGGGCGATGATCCCAAAGTAG |
| Anti-inflammatory | ANXA1 | fw | GGAGCTATCCCCATCTTCGC |
|  |  | rv | TGGCTTGCTTGTAGCACACT |
|  | CD206 | fw | GGGCTTGTGGGATGTCTTGA |
|  |  | rv | CTTCTGCCCAGTGTTTGCAC |
|  | CD209 | fw | GGTTCCGGAGTCTGACTGAAGTT |
|  |  | rv | GGTCAGGCGCTGTAGGATCTC |
|  | IL-10 | fw | CATGGGCCTGACATCAAGGA |
|  |  | rv | CGGAGGGTCTTCAGCTTCTC |
|  | TGF-β | fw | AAAAGAACTGCTGTGTTCGTCA |
|  |  | rv | GACCTTGCTGTACTGTGTGTCC |
|  | Galectin 3 | fw | CCTTCTGGACCCCTGAATGTG |
|  |  | rv | AGTCTGTTCGCATTGGGCTT |
| Immunomodulatory | SOCS 3 | fw | AGAGCGGCTTTTACTGGAGC |
|  |  | rv | CTGAGGGTGAAGAAGTGGCG |
|  | CD68 | fw | AGGCAGTGCAGTGGACATTC |
|  |  | rv | CAAGGTGTAAAGCTGGTGAAAC |
| Reference genes | HPRT 1 | fw | AGCTGGCTCCGTTATGGC |
|  |  | rv | ATCATCGCTAATCACCACGC |
|  | OvRPS 15 | fw | CGAGATGGTGGGCAGCAT |
|  |  | rv | GCTTGATTTCCACCTGGTTGA |
|  | YWHAZ | fw | TGAACTCCCCTGAGAAAGCC |
|  |  | rv | TCCGATGTCCACAATGTCAAGT |

**Supplementary Figure 1: Volcano plots of RNA sequence analysis of cortical microglia.** (A) Sham-Saline vs LPS-Saline showing 51 significantly downregulated and 55 significantly upregulated genes (B) Sham-Saline vs Sham-MAPC showing 33 significantly downregulated and 53 significantly upregulated genes, (C) LPS-Saline vs LPS-MAPC showing 314 significantly downregulated and 93significantly upregulated genes and (D) Sham-MAPC vs LPS-MAPC showing 115 significantly downregulated and 42 significantly upregulated genes. The volcano plot labels only the top 20 significant genes with known gene names, while genes lacking annotation are indicated as ‘unknown’.

**Supplementary table 2: GO terms per cluster**

| **Ontology** | **GO ID** | **Adjusted p-value** |
| --- | --- | --- |
| **Cluster 1: Microglial reactivity** | | |
| Cellular response to stress | GO:0033554 | 7.5E-23 |
| Response to stress | GO:0006950 | 3.9E-16 |
| Immune system process | GO:0002376 | 4.0E-09 |
| Proteolysis | GO:0006508 | 5.2E-09 |
| Cellular response to stimulus | GO:0051716 | 2.9E-08 |
| Response to stimulus | GO:0050896 | 2.9E-06 |
| Lymphocyte activation | GO:0046649 | 5.2E-06 |
| Regulation of response to stimulus | GO:0048583 | 1.3E-05 |
| Immune response | GO:0006955 | 7.2E-05 |
| B cell activation | GO:0042113 | 1.1E-04 |
| Leukocyte activation | GO:0045321 | 1.2E-04 |
| Regulation of cellular response to stress | GO:0080135 | 1.5E-04 |
| Innate immune response | GO:0045087 | 2.0E-04 |
| Regulation of immune system process | GO:0002682 | 2.1E-04 |
| Positive regulation of immune system process | GO:0002684 | 3.6E-04 |
| Regulation of response to stress | GO:0080134 | 6.7E-04 |
| Regulation of lymphocyte activation | GO:0051249 | 1.3E-03 |
| T cell activation | GO:0042110 | 1.6E-03 |
| Response to cytokine | GO:0034097 | 8.4E-03 |
| Positive regulation of response to stimulus | GO:0048584 | 8.9E-03 |
| Regulation of leukocyte activation | GO:0002694 | 9.1E-03 |
| Defense response | GO:0006952 | 1.0E-02 |
| Antigen processing and presentation | GO:0019882 | 1.1E-02 |
| Response to type II interferon | GO:0034341 | 1.3E-02 |
| Regulation of B cell activation | GO:0050864 | 1.5E-02 |
| Lymphocyte proliferation | GO:0046651 | 1.7E-02 |
| Regulation of cell activation | GO:0050865 | 2.0E-02 |
| Positive regulation of lymphocyte activation | GO:0051251 | 2.1E-02 |
| Regulation of immune response | GO:0050776 | 2.2E-02 |
| Positive regulation of leukocyte activation | GO:0002696 | 2.3E-02 |
| Regulation of lymphocyte proliferation | GO:0050670 | 2.6E-02 |
| Cellular response to type II interferon | GO:0071346 | 2.8E-02 |
| Regulation of apoptotic signaling pathway | GO:2001233 | 2.9E-02 |
| Cellular response to cytokine stimulus | GO:0071345 | 3.1E-02 |
| Leukocyte cell-cell adhesion | GO:0007159 | 3.5E-02 |
| Positive regulation of apoptotic process | GO:0043065 | 3.6E-02 |
| Positive regulation of immune response | GO:0050778 | 3.8E-02 |
| Cellular response to molecule of bacterial origin | GO:0071219 | 3.8E-02 |
| Antigen processing and presentation of peptide or polysaccharide antigen via MHC class II | GO:0002504 | 5.0E-02 |
| **Cluster 2: Metabolic processes** | | |
| Regulation of primary metabolic process | GO:0080090 | 1.0E-22 |
| DNA metabolic process | GO:0006259 | 3.7E-18 |
| Regulation of nucleobase-containing compound metabolic process | GO:0019219 | 5.6E-18 |
| Regulation of metabolic process | GO:0019222 | 2.8E-17 |
| Positive regulation of metabolic process | GO:0009893 | 5.6E-16 |
| Regulation of macromolecule metabolic process | GO:0060255 | 7.5E-15 |
| Positive regulation of macromolecule metabolic process | GO:0010604 | 2.1E-14 |
| Regulation of RNA metabolic process | GO:0051252 | 1.1E-12 |
| Negative regulation of nucleobase-containing compound metabolic process | GO:0045934 | 1.6E-09 |
| Protein metabolic process | GO:0019538 | 6.0E-09 |
| Positive regulation of nucleobase-containing compound metabolic process | GO:0045935 | 1.5E-08 |
| Negative regulation of RNA metabolic process | GO:0051253 | 4.7E-08 |
| Regulation of protein metabolic process | GO:0051246 | 1.6E-07 |
| Regulation of DNA metabolic process | GO:0051052 | 2.4E-07 |
| Positive regulation of DNA metabolic process | GO:0051054 | 7.7E-06 |
| Negative regulation of metabolic process | GO:0009892 | 1.6E-05 |
| Positive regulation of RNA metabolic process | GO:0051254 | 6.8E-05 |
| Negative regulation of protein metabolic process | GO:0051248 | 4.5E-02 |
| **Cluster 3: Cellular clearance** | | |
| Response to stress | GO:0006950 | 2.2E-07 |
| Mitochondrion organization | GO:0007005 | 1.0E-06 |
| Process utilizing autophagic mechanism | GO:0061919 | 5.4E-05 |
| Autophagy | GO:0006914 | 5.4E-05 |
| Cellular response to stress | GO:0033554 | 1.7E-04 |
| Cellular localization | GO:0051641 | 2.0E-04 |
| Response to stimulus | GO:0050896 | 4.0E-04 |
| Positive regulation of cellular process | GO:0048522 | 4.6E-04 |
| Macroautophagy | GO:0016236 | 8.3E-04 |
| Regulation of immune response | GO:0050776 | 1.3E-03 |
| Positive regulation of response to stimulus | GO:0048584 | 2.0E-03 |
| Positive regulation of immune response | GO:0050778 | 3.6E-03 |
| Endocytosis | GO:0006897 | 7.2E-03 |
| Regulation of autophagy | GO:0010506 | 1.4E-02 |
| Positive regulation of autophagy | GO:0010508 | 1.7E-02 |
| Immune response | GO:0006955 | 1.7E-02 |
| Response to biotic stimulus | GO:0009607 | 2.0E-02 |
| Immune system process | GO:0002376 | 2.6E-02 |
| Positive regulation of immune system process | GO:0002684 | 2.8E-02 |
| Activation of immune response | GO:0002253 | 4.3E-02 |
| Regulation of response to endoplasmic reticulum stress | GO:1905897 | 4.8E-02 |
| **Cluster 4: Biological regulation** | | |
| Biological regulation | GO:0065007 | 2.6E-03 |
| Regulation of biological process | GO:0050789 | 4.7E-03 |
| Regulation of cellular process | GO:0050794 | 1.3E-02 |

**Supplementary Figure 2: mRNA expression of immunoregulatory (a.), anti-inflammatory (b.) and pro-inflammatory (c.) mediators in white matter (WM) and cortex (Cx) microglia.** Data are shown as mean ± SEM. Group sizes were as follows: Sham-Saline (n=3), Sham-MAPC (n=4), LPS-Saline (n=5) and LPS-MAPC (n=6). Summary of the ANOVA results are presented on each panel. Results of the post-test are indicated with *p < 0.05. LPS=lipopolysaccharide, SAL=saline, MAPC=Multipotent adult progenitor cells.
